# A review of multi-omics data integration through deep learning approaches for disease diagnosis, prognosis, and treatment

**DOI:** 10.3389/fgene.2023.1199087

**Published:** 2023-07-20

**Authors:** Jael Sanyanda Wekesa, Michael Kimwele

**Affiliations:** School of Computing and Information Technology, Jomo Kenyatta University of Agriculture and Technology, Nairobi, Kenya

**Keywords:** deep learning, multi-omics, data integration, noncoding RNA, complex disease

## Abstract

Accurate diagnosis is the key to providing prompt and explicit treatment and disease management. The recognized biological method for the molecular diagnosis of infectious pathogens is polymerase chain reaction (PCR). Recently, deep learning approaches are playing a vital role in accurately identifying disease-related genes for diagnosis, prognosis, and treatment. The models reduce the time and cost used by wet-lab experimental procedures. Consequently, sophisticated computational approaches have been developed to facilitate the detection of cancer, a leading cause of death globally, and other complex diseases. In this review, we systematically evaluate the recent trends in multi-omics data analysis based on deep learning techniques and their application in disease prediction. We highlight the current challenges in the field and discuss how advances in deep learning methods and their optimization for application is vital in overcoming them. Ultimately, this review promotes the development of novel deep-learning methodologies for data integration, which is essential for disease detection and treatment.

## Introduction

The study of complex biological processes is akin to an integrative approach that combines muti-omics data to examine interrelationships in the biomolecules. Omics data such as genomics, proteomics, transcriptomics, and metabolomics have widely been utilized to address biomedical problems including disease diagnosis, prognosis, and therapies (Subramanian et al., 2020). Particularly, non-coding transcripts, mainly miRNAs and lncRNAs have been linked to cancer and other complex biological processes such as immune cell development and disorders (Winkle et al., 2021). Therefore, network topology information extracted from multi-source data contributes to the identification of potential associations between biomolecules and diseases (Shi et al., 2022).

Several molecular technologies for wet-lab based studies have identified molecular genetics of complex disease diagnosis, prognosis and therapeutic implications. The molecular diagnostics tests are gene panel, gene signature panel, gene expression panel, and tests that examine DNA, RNA, and proteins (Ishida et al., 2023). These tests are usually conducted for risk assessment, differential diagnosis, prognosis, and prediction of treatment response. The results obtained from the wet-lab methods can be integrated with computational approaches for the interpretation of the results. The integration of experimental and modeling techniques provides deeper insights, a more accurate and dynamic estimate of efficacies of treatments (Cotner et al., 2023). The integration of the two approaches can be achieved through the use of *in vitro* microfluidic devices such as microfluidic used to mimic vascularization (Cotner et al., 2023).

Through artificial intelligence technologies, imaging, molecular, and cellular data, the process of disease detection and diagnosis is fastened. Non-coding RNA (ncRNA) association with diseases has been discovered to identify potential candidates for biologists to explore disease mechanisms and subsequently, drug discovery for treatment. Complex diseases including cardiovascular diseases, breast and lung cancer are associated with abnormal expression of ncRNAs such as lncRNAs. Therefore, the computational methods prioritize the discovery of potential ncRNA-disease associations by utilizing biological information such as genome location, and tissue specificity (Xuan, et al., 2019). Identifying these associations contribute to understanding the pathogenesis, diagnosis, and treatment of human diseases. Complementarily, differential gene expression has also been used for disease diagnosis (Zhang et al., 2022).

The existing methods for predicting associations between ncRNAs and diseases are broadly classified into network-based and machine-learning based methods. The network-based methods utilize heterogeneous networks such as lncRNA-disease, lncRNA-miRNA and miRNA-disease datasets with known associations. Conversely, machine-learning based methods predict potential associations by building models which are trained to improve accuracy using association data. Sammut et al. (2022) proposed the integration of clinical, digital pathology, genomic and transcriptomic profiles to predict breast cancer therapy response. A study termed multi-omics graph convolutional network (MOGONET) proposed a supervised classification framework based on multi-omics data types for biomedical classification (Wang et al., 2021). A multi-omics integration model based on graph convolutional network (GCN) was proposed to analyze and classify cancer subtypes (Li J et al., 2022). Wang and Chen (2022) predicted miRNA-disease associations based on lncRNA-miRNA interactions and convolution networks. Other machine learning based interaction prediction methods for biomolecules such as lncRNA-protein interaction include, PLRPIM, DRPLPI, GPLPI, and GAE-LGA (Wekesa et al., 2019; Wekesa et al., 2020a; Wekesa et al., 2020b; Gao et al., 2022; Yu H et al., 2022). Some researchers have also developed multi-omics integration tools such as CustOmics that implements deep learning to integrate high dimensional and heterogeneous data (Benkirane et al., 2023).

The main objective of this article is to explore the application of deep learning in disease diagnosis, prognosis and therapies. We examine deep learning architectures such as convolutional neural networks (CNN), feed forward networks, and recurrent neural networks (RNN). Additionally, the advantages, disadvantages and obstacles faced by deep learning-based methods and recommendations on how to overcome them are included in the review. Selection of methods and tools in this review is based on the integration of multiple datasets and availability of the method in a public repository or as a tool or package. The knowledge gaps in the integration of multi-omics data through deep learning approaches include incompleteness of molecular interactome, challenges in identifying genes within genetic association regions and limited applications to human diseases. Moreover, limited model interpretability is also a challenge that limits adoption of the models due to complex prediction mechanism of the deep learning models. This article is the first to systematically compare the performance of deep learning algorithms in disease diagnosis, prognosis and treatment. In the following sections, the database resources of the multi-omics datasets are provided, their description and the references. Further, we provide a detailed account of how the datasets are used in the deep and machine learning algorithms.

## Multi-omics data integration, interpretation and disease prediction

### Database resources

To understand the roles of ncRNAs to diseases, determining their interactions is the key. Several disease related ncRNAs databases have been developed. The databases are composed of a collection and integration of resources focusing on circRNAs as disease biomarkers, and lncRNAs/mRNAs/miRNAs interactions with diseases. The ncRNA related databases, their description and URL links are listed in Table 1.

**TABLE 1:** Multi-omics ncRNA-disease data repositories.

Data repository	Interactions	Description	URL
lncRNADisease Bao et al., (2018)	lncRNA and disease	This database integrates experimentally supported lncRNA-disease associations	http://www.cuilab.cn/lncrnadisease
Lnc2Cancer 3.0. Gao et al, (2020)	lncRNA, cirRNA, and disease	This is a manually curated database with lncRNA, circRNA, and human cancer associations	http://www.bio-bigdata.net/lnc2cancer
MNDR Ning et al., (2021)	ncRNA (miRNA, lncRNA, circRNA, piRNA, snoRNA), disease	The database contains experimental and predicted mammalian ncRNA-disease associations	http://www.rna-society.org/mndr
circRNADisease Zhao et al., (2018)	circRNA and disease	This is a manually curated database of experimentally supported circRNA and disease associations	http://cgga.org.cn:9091/circRNADisease/
CircR2Disease Fan et al., (2021)	circRNAs and diseases	A Web Server for experimentally validated circRNA–disease associations and its application	https://bio.tools/circR2Disease
LncR2metasta Zhang et al., (2021)	lncRNAs and cancer	A manually curated database, aims at providing a comprehensive resource of lncRNA deregulation in various cancer metastatic events	http://lncr2metasta.wchoda.com/

### Interpretation of multi-omics datasets

Multi-omics data gives multiple views of a problem that are aggregated into context (Maghsoudi et al., 2022). Performing representation learning to explore information from multiple views is a challenging problem. The machine and deep learning models rely on feature information extracted from unlabeled data. Several tools have been proposed to facilitate interpretation of molecular features derived from multi-omics datasets that contain the biology of diseases. The tools incorporate concepts such as loss functions meant for obtaining puissant feature learning and prediction ability. For instance, a meta-learning deep learning method termed DeepLIFT was recently proposed that implements cox hazard loss to improve performance, intelligibility and interpretability of the model (Cho et al., 2023). Meta-learning, a learning-to-learn method, based on back propagation and cox hazard loss trained on transcriptomics, proteomics, and clinical datasets showed better performance than direct and transfer learning-based models.

Contrastive learning via data augmentation and other strategies and frameworks have attracted attention vastly in multi-omics data analysis. Contrastive learning is a self-supervised instance-level discriminative method that learns latent information with the aim of pulling the different views closer (Cui et al., 2023). Adapting algorithms that incorporate contrastive loss to evaluate cross-modal associations between datasets is paramount in understanding genetic modalities in the multi-omics data. Additionally, deep neural network algorithms improve the learned representations through contrastive masking to model nonlinear relationships. Many researchers have been contrasting high-dimensional features in the attempt to explore discriminative information from multiple views. However, low-dimensional representation is ignored albeit the significance of the information in the learned representation on downstream tasks. Li et al. proposed a method, regularized and hybrid Multiview coding (RHMC) a variant of contrastive learning method for comprehensive modeling of consistent information between multiple views (Li X et al., 2022). The method obtrudes global alignment on the learned representations in the latent space by computing the probability distribution of the views using the Wasserstein distance-based view alignment regularization. Therefore, contrastive learning learns high-quality representations and effectively supports multi-omics integration (Yang et al., 2022).

**FIGURE 1:**
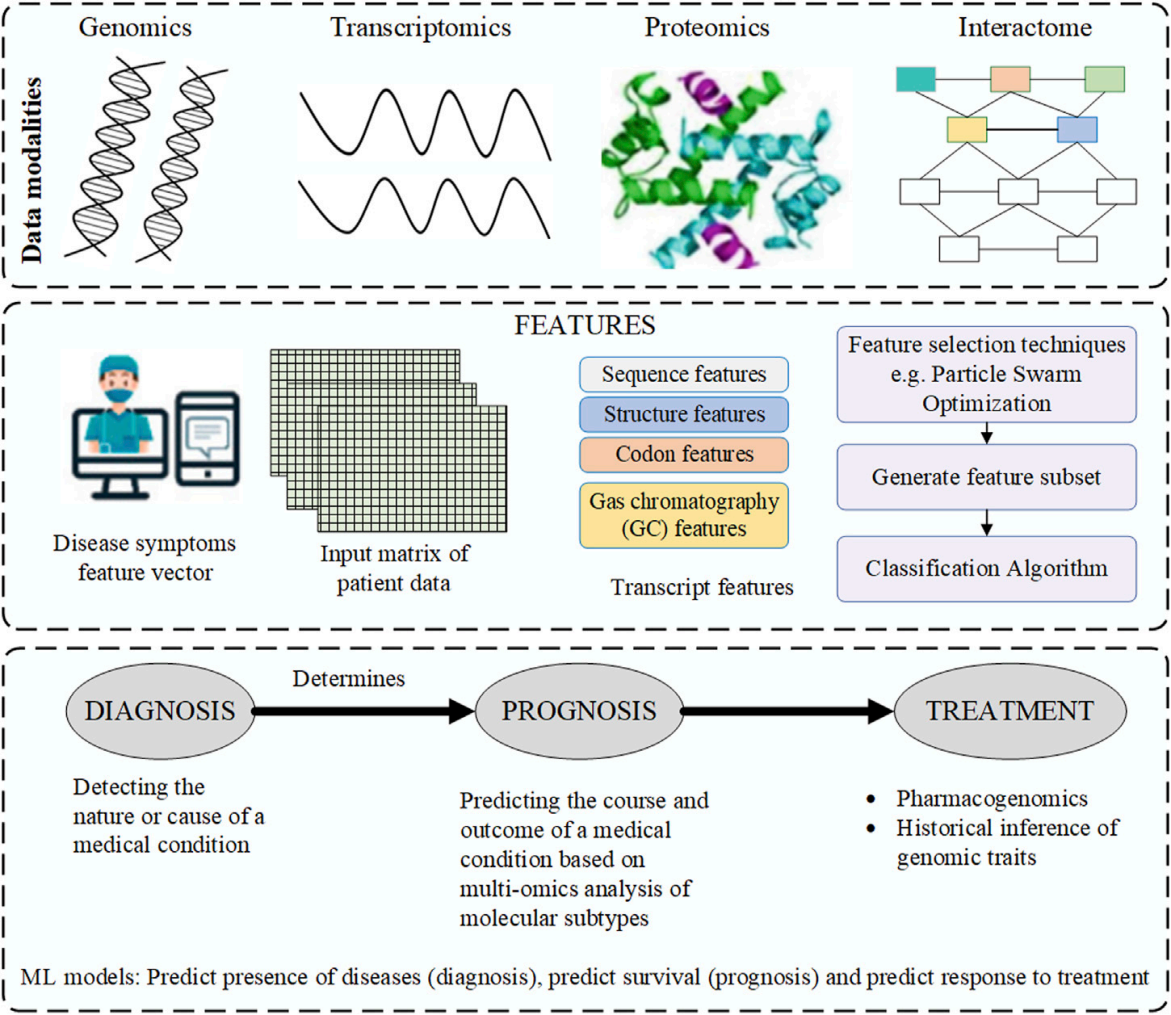
The data modalities used by machine learning (ML) prediction models for diagnosis, prognosis and treatment.

Comprehensive understanding of human complex diseases requires the analysis of multi-omics data reciprocally with clinical information. Based on the analysis, useful insights into the cellular functions are derived. The integration helps to understand the interplay of the biomolecules and in the assessment of the information from the omics data. Further, it improves prognostic and predictive accuracies hence better treatment and prevention of the diseases (Subramanian, et al., 2020). The gap between genotype and phenotype is traversed through the flow of information from one omics level to the other. However, challenges associated with data integration arise from complexity of the data and difficulty in the interpretation of the analysis results. There are platforms such as GraphOmics for exploring and integrating multiple omics datasets and also used for hypothesis generation (Wandy and Daly, 2021). The goal of the platforms is to uncover associations between unknown entities not captured in the knowledge base and methods such as correlation analysis and other analysis methods. A hybrid multi-omics network from longitudinal multi-omics data was proposed to facilitate the interpretation of the data. The method provides interpretation guidelines to explore network generated from multi-omics data to highlight inter and intra omics mechanisms and interactions (Bodein et al., 2021). Figure 1 below is an illustration of the data multimodalities and how the machine learning prediction models are used to predict diseases.

### Prediction methods based on deep learning

Disease diagnosis is primarily based on the patient history and physical examination done by health experts. It is often a difficult task due to the complexity of the disease mechanism and ambiguity of the symptoms that require proper diagnostic procedures. Moreover, a series of medical tests are needed which are expensive and because of human error patients may be misdiagnosed. Therefore, artificial intelligence develops algorithms and techniques for correct disease diagnosis, prognosis, and treatment. Machine learning (ML) is a subset of artificial intelligence that is based on mathematical and statistical approaches. The ML based diagnosis methods are developed based on healthcare data such as X-ray, MRI and tabular data with patients’ conditions, age, gender, body mass index (BMI) and blood pressure (BP). Other features used for prediction include genetic features and interaction network-based features. The features are fed into the algorithm which is able to generalize the knowledge based on a pattern observed from a group of samples. Generally, the immense growth of ML over the years is attributed to the advancement of technology and availability of data generated by academics and practitioners (Ahsan et al., 2022).

Deep learning models are effective for decoding pathological images, interaction and prognosis prediction. Several machine and deep learning algorithms have been used for breast cancer detection namely support vector machine (SVM), CNN, bidirectional RNN (BiRNN) and naïve bayes (Vaka et al., 2020). It has been proven that the expression levels of ncRNAs are altered in cancer cells or tumor tissues. Therefore, research on the expression of ncRNAs under pathological conditions is valuable for identification of novel biomarkers and target therapeutics. A biomarker is a molecule that is relatively easy to detect and offers credible information on diagnosis, prognosis and other disease parameters (Volovat et al., 2020). The implementation of the models is done through platforms such as Tensorflow, PyTorch, and Caffe (Shoaib et al., 2023). Tensorflow and PyTorch are open-source libraries for computation via creation of dataflow graphs and distributed training respectively. Caffe is an open-source deep learning framework for video and image classification.

Yu Z et al. (2022) published a review paper on popular deep learning algorithms for disease prediction. One of the popularly used deep learning model is CNN which is mostly suitable for learning image features. The model combines local receptive field, shared weight and down sampling. Receptive field in the convolutional kernel extracts visual features such as edges and corners. Shared weight feature is realized through scanning of the images by the convolution kernel using the same weight. One limitation of CNN is large amount of labeled data required to train the model. In a study conducted by Khan et al. deep CNN model was trained and used to classify normal and abnormal breast tissue (Heenaye-Mamode Khan et al., 2021). Digital images and health records of women were used to train and test the proposed model. The model predicted biopsy malignancy and differentiated normal from abnormal screening examinations. The features extracted include binding site, morphological and genetic features. Other deep learning models include LSTM for learning sequence-based features, stacked autoencoder for dimensionality reduction and classification and deep belief network. Factorization machine deep learning (FMDNN) was proposed to solve the problems of DNN (Yu et al., 2021). This model learns low and high order feature interactions. Factorization machine (FM) eliminates pre-training and facilitates an end-to-end training of the neural network. An FM based neural network termed DeepFM was proposed to predict the presence or absence of hepatitis (Yu et al., 2021).

Graph neural networks (GNNs) operate on graph-structured data and have successfully been used in network biology applications. Hernández-Lorenzo et al. (2022) proposed an Alzheimer’s disease prediction model through Graph Neural Networks. The study presented a genotype-to-phenotype prediction pipeline that uses GNNs in combination with protein-protein Interaction (PPI) and functional biological networks. Huang and Chung (2022) proposed edge-variational graph convolutional networks (EV-GCN) for prediction of Autism Spectrum Disorder, Alzheimer’s disease and ocular disease. Further, Monte-Carlo edge dropout uncertainty estimation was implemented to estimate the predictive uncertainty related to the constructed graph. EV-GCN, a population-based disease analysis method uses multi-modal medical data to evaluate the proposed method. In another recent study, a weighted-link GNN algorithm that combined graph auto-encoder and graph convolutional network was put forward (Cheng et al., 2023). The algorithm produced the best classification performance in the lung cancer knowledge classification compared to other state-of-the-art methods. From the highlighted GNN based methods, it is observed that the algorithms extract meaningful features which enable them to achieve superior performance. The methods are knowledge guided such that they inject knowledge from a graph structure medical ontology into deep models via attention mechanisms. Despite the advantages of GNNs, their limitations include scalability such that it is difficult to scale the edges of graphs based on the type and relations. Table 2 consists of recently proposed prediction models, the datasets used and the task performed by the models.

**TABLE 2:** Deep learning-based disease prediction methods.

Model	Dataset	Sample size	Task/Use-case	References
DPGNI	Gene network	1,728 diseases	Disease Classification	Mi, et al. (2019)
MIDDM	Infectious diseases medical records	20,620 cases, 7 infectious diseases	Multi- disease Classification	Wang M et al. (2022)
HCNN	Medical images	954 normal and disease sub-types	Disease Classification	An, et al. (2021)
DOCTOR	Chest radiographs, eye-gaze coordinates	1083 chest radiographs	Disease Classification	Watanabe, et al. (2022)
DTLC	Chest CT	852 infected patients images	Disease classification	Pathak, et al. (2022)
MAGCN	lncRNA, miRNA, disease	10,465 LMIs and 11,253 MDAs	Interaction prediction	Wang and Chen (2022)
SGAEMDA	miRNA, disease	5,430 associations of 383 complex diseases and 495 miRNAs	Interaction prediction	Wang S et al. (2022)
MVIFMDA	miRNA, disease	12,446 associations of 853 miRNAs and 591 diseases	Interaction prediction	Xie X et al. (2022)
IGNSCDA	circRNA, disease	612 associations of 533 circRNAs and 89 diseases	Interaction prediction	Lan, et al. (2021)
MNNMDA	Microbe, disease	9,660 associations of 2,546 microbes and 537 diseases	Interaction prediction	Liu Z et al. (2023)

### Prediction methods based on biological molecular network

The association between multi-omics data, and diseases has been extensively explored by researchers. It has been established that multi-omics data can effectively predict the diagnosis, prognosis, and treatment of diseases (Pan et al., 2022). The application of computational biology tools to integrate omics data to investigate disease pathogenesis is known as network medicine. The analytical methods in network medicine based on molecular networks exploit protein-protein interaction, correlation-based networks, gene regulatory networks and Bayesian networks (Silverman et al., 2020). The network-based approaches exploit graph-theoretic (random walks, network propagation and path search), machine and deep learning. Analyzing topology of the network nodes uncovers specificities and similarities in how genes play regulatory roles and draws insights on diseases similarities.

Disease associated genes prediction can be accomplished through graph-theoretic algorithms, machine learning algorithms and their integration. This task is based on the assumption that diseases linked to the same genes are closely located in a molecular network such as PPI, co-expression networks and gene regulatory networks. PPI is the regularly used network among the three, this is attributed to the factuality that interacting proteins perform common biological functions. While gene expression refers to the process of converting genetic information into functional RNA or protein, gene regulation is the process of controlling the expression of genes. In a gene regulatory network, the edges represent not only interaction but also other biological processes such as reaction, activation or inhibition. A study by Hasankhani et al. (2021) revealed that through the integration of co-expression networks based on the hub genes and PPI networks, key hub-high traffic genes were identified as potential therapeutic targets for COVID-19 pandemic. In 2022, a study was done to predict gastric cancer diagnosis, prognosis, and drug repurposing based on gene expression signatures. The study used gene expression datasets to predict novel diagnostic candidates. Recently, co-expression network analysis of down syndrome was conducted to explore cell types associated with abnormal brain development (Seol et al., 2023). Through cell-type enrichment analysis on gene expression modules, gene modules associated with specific brain types were identified and functional annotation provided insights into the role of specific cell types in biological processes.

Wu et al. (2013) proposed qNABpredict, a taxonomy-agnostic model that predicts content of the nucleic acid-binding residues. The tool is designed to predict details of protein-NA (nucleic acid) interactions for large protein families and proteomes. Interactions between proteins and nucleic acids from protein sequences are critical in a wide range of cellular functions such as gene expression and regulation. Discovery of biomarkers through differential expression and molecular associations is a focal point of research. Technological advancements in molecular analysis have enabled identification of a large number of candidate biomarkers for complex diseases. Biomarkers can be used to determine disease stage in disease diagnosis. Additionally, they are used to assess the efficacy and monitor the response to new drugs or therapeutic intervention.

### Emerging technologies and case studies

**FIGURE 2:**
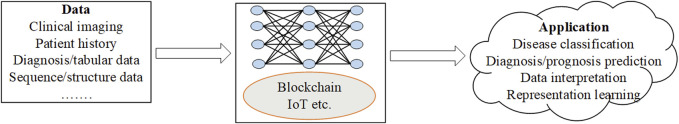
Application of deep learning with current technologies in computational biology.

The profound advancements in technology assist in the development of decision support systems that provide accurate and reliable evidence-based solutions in different domains such as finance and medicine. In this section, we investigate prediction methods based on different types of diseases including breast cancer, brain cancer and hybrid disease detection. We examine emerging technologies such as blockchain, internet of things (IoTs), their evolution and integration with deep learning. Blockchain is a technology designed to offer high-level security, transparency and tamper proof data management for applications. It uses cryptographic signature that links blocks in the chain and generate unbroken chain of records. On the other hand, IoT based platforms are based on intelligent hardware, deep learning and mobile terminals to develop applications. By integrating blockchain technology and machine/deep learning, developed applications are able to extract valuable insights from data while preserving privacy. Figure 2 below shows the data sets, integration of deep learning with emerging technologies such as blockchain and IoT and their application in computational biology.

### Advanced technologies for breast cancer detection

Breast cancer is a major cause of mortality worldwide. Studies have shown that it emerges from abnormally replicated breast cells. Detection methods include mammography, CT, MRI, ultrasound and biopsy. Machine and deep learning models have been proposed to aid in the detection of the malignant breast cancer such as inflammatory, invasive among others. Internet medical of things (IoMT) is a recently proposed method for detection and management of breast cancer. The method was implemented through gated recurrent units (GRU) a recurrent neural network model (Aldhyani et al., 2023). The method employs blockchain technology using advanced encryption standard (AES) cryptosystem. A cloud health resource-sharing model based on consensus blockchain technology is a platform developed to perform breast tumor diagnosis (Zhu et al., 2019).

### Advanced technologies for brain cancer detection

Imaging technology provides an interior anatomy of patients that assist in the detection of abnormal tissues. Brain tumor, causes impairment and death in both men and women. Diagnosis of brain cancer is usually based on studying MRI scans which is laborious, error-prone and time-consuming. Therefore, deep learning models in conjunction with IoT and blockchain technology offer fast, secure, and precise prediction mechanism. Several authors have proposed classifiers that utilize the two technologies. An adaptive neuro-fuzzy system classifier for detecting brain tumor implemented with IoT through simulations was developed by (Sandya et al., 2023). A deep learning method for brain tumor detection based on blockchain technology was proposed to predict using MRI images (Mohammad et al., 2023).

### Advanced deep learning-based models for hybrid disease detection

Hybrid disease detection systems are models that diagnose multiple ailments. A blockchain-based multi-diagnosis deep learning model for disease classification was recently proposed to provide security of data shared in the healthcare sector (Rahal et al., 2023). The approach combines data from multiple sources for disease diagnosis by training and testing deep learning models on breast cancer, lung cancer and diabetes datasets. Another study based on federated learning, blockchain technology and deep learning models was proposed for classification of four respiratory diseases, COVID-19, Pneumonia, Tuberculosis, and Lung Opacity (Noman et al., 2023). The model is a web-based real-time classification tool.

### Experimental evaluation

This article is a qualitative research comprising of recent studies on the prediction of complex disease diagnosis, prognosis and treatment using multi-omics data and emerging technologies. To effectively measure the prediction models performance, cross validation system which circumvents cross-section prejudice is applied. Five-fold and ten-fold cross-validation techniques are most commonly used to assess classifiers performance. In the cross-validation techniques, the dataset is randomly fragmented into training and testing sets. To quantitatively appraise the efficacy of the classifiers, the evaluation metrics include accuracy, Mathew’s correlation coefficient (MCC), precision, recall, specificity and area under the curve (AUC). Accuracy measures the ratio of correct predictions over all the samples. Precision indicates the ratio of correctly predicted positive samples over all the predicted positive samples. Recall indicates the ratio of correctly predicted positive samples over all the positive samples.

### Comparison of models

**FIGURE 3:**
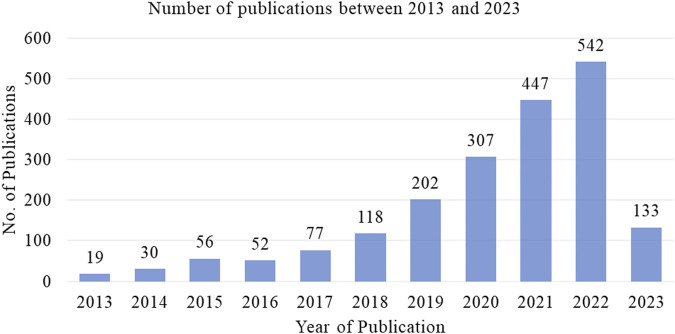
Number of publications on disease diagnosis based on machine-learning algorithms.

The number of publications on the computational prediction of diseases has steadily increased over time. The authors main aim has been to improve the performance through different approached including dimensionality reduction and feature selection mechanisms. Figure 3 illustrates a summary of the number of publications between 2013 and 2023, the information is obtained from Scopus data. Table 3 represents the studies’ findings and the respective references. The accuracies obtained by the methods is notably high ranging between 76% and 99%. Particularly, the performance of DRAE (Menagadevi et al., 2023) recorded an accuracy of 98%. The high performance is attributed to the pre-processing techniques implemented, modified optimal curvelet thresholding and Octagon histogram equalization. The two techniques removed noise from the datasets hence Then residual autoencoder architecture is used for extracting features and SVM implemented for classification. Moreover, it can be observed in Table 3 that the deep learning models performance were positively influenced by the optimization and feature normalization mechanisms they implemented. A checkmark in the third column indicates whether the model was designed to address the specific challenge. In the fourth column, the checkmark indicates implementation of heterogeneous or hybrid machine learning for disease prediction.

**TABLE 3:** Comparison of prediction methods.

Method	Description	Missing data	Het. ML	References
DCNN	A deep CNN with self-attention implemented to predict heart disease	✓		Arooj et al. (2022)
DPMLT	Ensemble machine learning model for multi-disease predicting	✓	✓	Park et al. (2021)
NN with VGG16	A deep learning model for Pneumonia prediction from Chest X-Ray Images using VGG-16 and Neural Networks			Sharma and Guleria (2023)
PDLM	Classification of breast cancer using pre-trained deep learning		✓	Kadry et al. (2023)
MNC-Net	A multi-task graph structure learning based on node clustering for early Parkinson’s disease diagnosis		✓	Huang et al. (2023)
Eadn	Autoencoder based method for detection of Parkinson’s disease	✓	✓	Rao (2023)
DRAE	Deep residual autoencoder for Alzheimer disease prediction		✓	Menagadevi et al. (2023)
SCAN	Bayesian variational autoencoder for breast cancer prognosis prediction	✓	✓	Hsu and Lin (2023)
RGNN	Recurrent neural network and graph neural network for next-period (medical event prediction) prescription classification		✓	Liu et al. (2020)

### Leveraging multi-omics data for disease related insights

Genome, transcriptome, proteome and other omics data collaborate to perform complex cellular processes. Some researchers have proven that multi-omics datasets help to unravel the molecular mechanisms. The cancer genome atlas (TCGA) data identify distinct molecular subtypes of cancer with the aim of improving diagnostic methods, treatment standards, and finally to prevent cancer (Tomczak, et al., 2015). The data combines DNA methylation, mRNA/microRNA expression and proteomics data (reverse-phase protein arrays). The analysis of the datasets output a comprehensive catalog of genetic and epigenetic drivers of cancer e.g., breast cancer subtypes (Koboldt, et al., 2012).

Aside from disease diagnosis, multi-omics data has been effectively used for precise cancer treatment. Chai et al. (2021) integrated multi-omics data for accurate cancer prognosis prediction. The method implemented denoising autoencoder for robust representation of multi-omics data to estimate patient’s risk of cancer through the Cox proportional hazard model. Another model named MSDLM was recently proposed to predict prognosis and therapy response in colorectal cancer (Foersch, et al., 2023). The model was developed based on cellular patterns of anti-tumor immunity and was determined to outperform clinical, molecular and immune cell-based parameters.

The biggest challenge in training models is data quality. Particularly in the application of deep learning models in disease related predictions, high quality medical data is of great significance. However, the quality of medical data is of low quality despite the availability of the data in large quantity. The problems include, the need for medical expertise to label samples, abnormal features and mismatch between the training and actual data samples. To circumvent the challenges of low amount and quality of image, speech and text data, techniques like up sampling, Fourier transform and augmentation are used to improve the quality. Moreover, attention mechanisms are implemented to improve representation ability and interpretability.

Feature selection entails the selection of the most relevant features during model construction. Some algorithms have built-in feature selection methods and penalization mechanisms for reducing overfitting while others rely on the features fed into them. An exhaustive search strategy is usually implemented on the feature subsets to identify the most optimal features suitable for the task. The search strategies include filter methods such as Chi-square and linear discriminant, wrapper methods such as genetic algorithms and embedded methods such as least absolute shrinkage and selection operator (LASSO) and ridge regression. The benefits of feature selection include reduced model training time, increased performance and Feature selection is also a means of achieving dimensionality reduction which has advantages such as complexity reduction. Models that rely on knowledge graph information with historical information and domain knowledge have been found to have high accuracy on diagnosis prediction tasks. To leverage the knowledge graphs, an end-to-end robust solution can be achieved through the features of GNN algorithm. Also, Huang et al. proposed combining graph structure learning and multi-task representation learning through multi-task node cluster to address the challenge of model interpretability (Huang et al., 2023).

### Challenges, recommendations and future prospects

The main challenge in disease diagnosis, prognosis, and treatment is medical uncertainty which affects both human (physicians) and deep learning models implementation in healthcare. This is a scenario whereby, there is no definite solution to a symptom presented by a patient for diagnosis. In this regard, mathematical and statistical models have been used to describe the mechanisms and dynamics of biological experimental findings and the degree of uncertainty quantified. Several deep learning models that quantify uncertainty in the classification results have been proposed including (Arco et al., 2023; Cifci 2023; Ren et al., 2023). Therefore, collaboration between computational biology experts who develop the prediction models with medical professionals to test the proposed models in real clinical scenarios is highly recommended. Thus, findings from the computational prediction models should be verified using wet-lab experiments and extensive pathway analysis. To overcome the shortcomings of the computational and biological experiments, firstly, the quantitative experimental methods can be used to measure dynamics of the diseases *in vitro*. Then, the wet-lab studies and data generated from them can be integrated with the mathematical modeling approaches for more accurate and context-based interpretation of the results.

Deep learning algorithms have been the most promising computational models for multi-omics data integration analysis. The models have achieved great success due to their superior feature representation capability and the end-to-end training paradigm. Generating correlations between the omics data is of utmost significant (Gong et al., 2023). Other challenges encountered in the algorithm implementation include overfitting, inequality, poor interpretability, privacy protection, and lack of reliable validation. To address model overfitting, feature selection and deep learning based multimodal feature fusion has successfully been implemented. Moreover, legal and ethical challenges in the implementation of deep learning models in healthcare is a major problem due to the use of personal information which can cause harm to the people involved through exposing them to discrimination etc. The different ways of ensuring protection of data are ethical guidelines, transparency and explainability, robustness, privacy, and accountability. Practically, blockchain technologies have been proposed to deal with the privacy protection as a means of boosting trust in technology. To solve reliable validation techniques challenge, methods such as cross-validation a resampling technique is used. To further enhance the performance of deep learning algorithms, deeper and wider neural networks with many layers and channels is crucial to cater for the depth of representation.

Attention is an algorithm that suppresses irrelevant information and accentuates relevant information. Adding attention mechanisms to neural network algorithms such as CNN enhances network performance and it is also used for dimensionality reduction. It is implemented during feature extraction process. Attention mechanisms dynamically assigns weights to the features to minimize the effect of less important features. The variants of attention mechanisms are self-attention (Arooj et al., 2022), graph attention (Wekesa et al., 2020a), coordinate attention (Xie C et al., 2022), dimensionality reduction attention (Wang and Wang, 2022), residual attention (Zhao et al., 2022), and spatial attention. Self-attention enhances information content by focusing on a single sequence to compute the sequence representation. Coordinate attention mechanism embeds position/location information in the feature map to enable the network to focus on important regions. Dimensionality reduction attention mechanism aims at limiting skewness error during feature extraction. Combining the attention mechanisms such as spatial and residual significantly improves the algorithm performance through broadening the representation power of the baseline network in a classification problem.

Researchers are developing learning paradigms such as meta-learning categorized as metric (similarity based on distance metrics), model (internal and external memory) and optimization (optimizing model parameters for fast learning). Meta-learning helps to solve data scarcity problems in disease diagnosis (Liu H et al., 2023). Multi-diagnosis methods are based on the three dimensions of meta-learning. Since deep learning algorithms are applicable to data-intensive learning tasks, integration of knowledge representation and reasoning in the development of complex systems is an area that is yet to be widely explored. Knowledge representation and reasoning is applicable in medical robotics and other domains. For instance, Ontology for Robotic Orthopedic Surgery (OROSU) is a robot used to perform surgical procedures (Gonçalves and Torres, 2015). Such systems require ethical procedures to be adhered to for their adoption to be acceptable in practice.

## Conclusion

Complex molecular networks are involved in human diseases. To fully elucidate the molecular system and understand biological processes involved in diseases, the dynamic dimensions of biological information is critical. This paper presents a survey on algorithmic frameworks developed to unravel the significance of multi-omics in disease classification, diagnosis, prognosis and treatment. Diseases that have been explored include cancer, Alzheimer and down syndrome among others. We provide a comprehensive summary of the databases of omics data and discus the challenges facing the implementation. Our review found that deep learning models achieve the level of accuracy in medical diagnostics prognosis similar to healthcare professionals. The challenges of deep learning such as complexity in the models need to be addressed to improve the quality and interpretability of future studies.

## References

[B1] Ahsan M. M. Luna S. A. Siddique Z. (2022). “Machine-learning-based disease diagnosis: A comprehensive review,” in Healthcare. Basel, Switzerland: MDPI, 541. 10.3390/healthcare10030541 PMC8950225 35327018

[B2] Aldhyani T. H. Khan M. A. Almaiah M. A. Alnazzawi N. Hwaitat A. K. A. Elhag A. (2023). A secure internet of medical things framework for breast cancer detection in sustainable smart cities. Electronics 12 (4), 858. 10.3390/electronics12040858

[B3] An G. Akiba M. Omodaka K. Nakazawa T. Yokota H. (2021). Hierarchical deep learning models using transfer learning for disease detection and classification based on small number of medical images. Sci. Rep. 11 (1), 4250. 10.1038/s41598-021-83503-7 33649375 PMC7921640

[B4] Arco J. E. Ortiz A. Ramírez J. Martínez-Murcia F. J. Zhang Y. D. Górriz J. M. (2023). Uncertainty-driven ensembles of multi-scale deep architectures for image classification. Inf. Fusion 89, 53–65. 10.1016/j.inffus.2022.08.010

[B5] Arooj S. Rehman S. U. Imran A. Almuhaimeed A. Alzahrani A. K. Alzahrani A. (2022). A deep convolutional neural network for the early detection of heart disease. Biomedicines 10 (11), 2796. 10.3390/biomedicines10112796 36359317 PMC9687844

[B6] Bao Z. Yang Z. Huang Z. Zhou Y. Cui Q. Dong D. (2018). LncRNADisease 2.0: An updated database of long non-coding RNA-associated diseases. Nucleic Acids Res. 47 (D1), D1034–D1037. 10.1093/nar/gky905 PMC6324086 30285109

[B7] Benkirane H. Pradat Y. Michiels S. Cournède P. H. (2023). CustOmics: A versatile deep-learning based strategy for multi-omics integration. PLoS Comput. Biol. 19 (3), e1010921. 10.1371/journal.pcbi.1010921 36877736 PMC10019780

[B8] Bodein A. Scott-Boyer M. P. Perin O. Lê Cao K. A. Droit A. (2021). Interpretation of network-based integration from multi-omics longitudinal data. Nucleic Acids Res. 50 (5), e27. 10.1093/nar/gkab1200 PMC8934642 34883510

[B9] Chai H. Zhou X. Zhang Z. Rao J. Zhao H. Yang Y. (2021). Integrating multi-omics data through deep learning for accurate cancer prognosis prediction. Comput. Biol. Med. 134, 104481. 10.1016/j.compbiomed.2021.104481 33989895

[B10] Cheng C.-H. Ji Z.-T. Baker M. Weiss J. W. (2023). Racial/ethnic and gender disparities in perceived stress and physical activity in college. Appl. Intell., 1–7. 10.1080/07448481.2022.2155461 36595637

[B11] Cho H. J. Shu M. Bekiranov S. Zang C. Zhang A. (2023). Interpretable meta-learning of multi-omics data for survival analysis and pathway enrichment. Bioinformatics 39, btad113. 10.1093/bioinformatics/btad113 36864611 PMC10079355

[B12] Cifci M. A. (2023). A deep learning-based framework for uncertainty quantification in medical imaging using the DropWeak technique: An empirical study with baresnet. Diagnostics 13 (4), 800. 10.3390/diagnostics13040800 36832288 PMC9955446

[B13] Cotner M. Meng S. Jost T. Gardner A. De Santiago C. Brock A. (2023). Integration of quantitative methods and mathematical approaches for the modeling of cancer cell proliferation dynamics. Am. J. Physiology-Cell Physiology 324 (2), C247–C262. 10.1152/ajpcell.00185.2022 PMC9886359 36503241

[B14] Cui W. Bai L. Yang X. Liang J. (2023). A new contrastive learning framework for reducing the effect of hard negatives. Knowledge-Based Syst. 260, 110121. 10.1016/j.knosys.2022.110121

[B15] Fan C. Lei X. Tie J. Zhang Y. Wu F. X. Pan Y. (2021). CircR2Disease v2.0: An updated web server for experimentally validated circRNA–disease associations and its application. Genomics, Proteomics Bioinforma. 20, 435–445. 10.1016/j.gpb.2021.10.002 PMC9801044 34856391

[B16] Foersch S. Glasner C. Woerl A. C. Eckstein M. Wagner D. C. Schulz S. (2023). Multistain deep learning for prediction of prognosis and therapy response in colorectal cancer. Nat. Med. 29 (2), 430–439. 10.1038/s41591-022-02134-1 36624314

[B17] Gao Y. Shang S. Guo S. Li X. Zhou H. Liu H. (2020). Lnc2Cancer 3.0: An updated resource for experimentally supported lncRNA/circRNA cancer associations and web tools based on RNA-seq and scRNA-seq data. Nucleic Acids Res. 49 (D1), D1251–D1258. 10.1093/nar/gkaa1006 PMC7779028 33219685

[B18] Gao M. Liu S. Qi Y. Guo X. Shang X. (2022). GAE-LGA: Integration of multi-omics data with graph autoencoders to identify lncRNA–PCG associations. Briefings Bioinforma. 23 (6), bbac452. 10.1093/bib/bbac452 36305456

[B19] Gonçalves P. J. S. Torres P. M. B. (2015). Knowledge representation applied to robotic orthopedic surgery. Robotics Computer-Integrated Manuf. 33, 90–99. 10.1016/j.rcim.2014.08.014

[B20] Gong P. Cheng L. Zhang Z. Meng A. Li E. Chen J. (2023). Multi-omics integration method based on attention deep learning network for biomedical data classification. Comput. Methods Programs Biomed. 231, 107377. 10.1016/j.cmpb.2023.107377 36739624

[B21] Hasankhani A. Bahrami A. Sheybani N. Aria B. Hemati B. Fatehi F. (2021). Differential Co-expression network analysis reveals key hub-high traffic genes as potential therapeutic targets for COVID-19 pandemic. Front. Immunol. 12, 789317. 10.3389/fimmu.2021.789317 34975885 PMC8714803

[B22] Heenaye-Mamode Khan M. Boodoo-Jahangeer N. Dullull W. Nathire S. Gao X. Sinha G. R. (2021). Multi-class classification of breast cancer abnormalities using Deep Convolutional Neural Network (CNN). PLoS One 16 (8), e0256500. 10.1371/journal.pone.0256500 34437623 PMC8389446

[B23] Hernández-Lorenzo L. Hoffmann M. Scheibling E. List M. Matías-Guiu J. A. Ayala J. L. (2022). On the limits of graph neural networks for the early diagnosis of Alzheimer’s disease. Sci. Rep. 12 (1), 17632. 10.1038/s41598-022-21491-y 36271229 PMC9587223

[B24] Hsu T.-C. Lin C. (2023). Learning from small medical data—Robust semi-supervised cancer prognosis classifier with bayesian variational autoencoder. Bioinforma. Adv. 3 (1), vbac100. 10.1093/bioadv/vbac100 PMC9832968 36698767

[B25] Huang Y. Chung A. C. (2022). Disease prediction with edge-variational graph convolutional networks. Med. Image Anal. 77, 102375. 10.1016/j.media.2022.102375 35144198

[B26] Huang L. Ye X. Yang M. Pan L. Zheng S. H. (2023). MNC-Net: Multi-task graph structure learning based on node clustering for early Parkinson’s disease diagnosis. Comput. Biol. Med. 152, 106308. 10.1016/j.compbiomed.2022.106308 36462371

[B27] Ishida C. Zubair M. Gupta V. (2023). “Molecular genetics testing,” in StatPearls (Treasure Island (FL): StatPearls Publishing LLC.). 32809547

[B28] Kadry S. Crespo R. G. Herrera-Viedma E. Krishnamoorthy S. Rajinikanth V. (2023). Classification of breast thermal images into healthy/cancer group using pre-trained deep learning schemes. Procedia Comput. Sci. 218, 24–34. 10.1016/j.procs.2022.12.398

[B29] Koboldt D. C. Fulton R. S. McLellan M. D. Schmidt H. Kalicki-Veizer J. McMichael J. F. (2012). Comprehensive molecular portraits of human breast tumours. Nature 490 (7418), 61–70. 10.1038/nature11412 23000897 PMC3465532

[B30] Lan W. Dong Y. Chen Q. Liu J. Wang J. Chen Y. P. P. (2021). Ignscda: Predicting CircRNA-disease associations based on improved graph convolutional network and negative sampling. IEEE/ACM Trans. Comput. Biol. Bioinforma. 19, 1. 10.1109/tcbb.2021.3111607 34506289

[B31] Li J. Qiang W. Zheng C. Su B. (2022). Rhmc: Modeling consistent information from deep multiple views via Regularized and Hybrid Multiview Coding. Knowledge-Based Syst. 241, 108201. 10.1016/j.knosys.2022.108201

[B32] Li X. Ma J. Leng L. Han M. Li M. He F. (2022). MoGCN: A multi-omics integration method based on graph convolutional network for cancer subtype analysis. Front. Genet. 13. 10.3389/fgene.2022.806842 PMC8847688 35186034

[B33] Liu S. Li T. Ding H. Tang B. Wang X. Chen Q. (2020). A hybrid method of recurrent neural network and graph neural network for next-period prescription prediction. Int. J. Mach. Learn. Cybern. 11 (12), 2849–2856. 10.1007/s13042-020-01155-x 33727983 PMC7308113

[B34] Liu H. Bing P. Zhang M. Tian G. Ma J. Li H. (2023). Mnnmda: Predicting human microbe-disease association via a method to minimize matrix nuclear norm. Comput. Struct. Biotechnol. J. 21, 1414–1423. 10.1016/j.csbj.2022.12.053 36824227 PMC9941872

[B35] Liu Z. Chen Y. Zhang Y. Ran S. Cheng C. Yang G. (2023). Diagnosis of arrhythmias with few abnormal ECG samples using metric-based meta learning. Comput. Biol. Med. 153, 106465. 10.1016/j.compbiomed.2022.106465 36610213

[B36] Maghsoudi Z. Nguyen H. Tavakkoli A. Nguyen T. (2022). A comprehensive survey of the approaches for pathway analysis using multi-omics data integration. Briefings Bioinforma. 23 (6), bbac435. 10.1093/bib/bbac435 PMC9677478 36252928

[B37] Menagadevi M. Mangai S. Madian N. Thiyagarajan D. (2023). Automated prediction system for Alzheimer detection based on deep residual autoencoder and support vector machine. Optik 272, 170212. 10.1016/j.ijleo.2022.170212

[B38] Mi Z. Guo B. Yin Z. Li J. Zheng Z. (2019). Disease classification via gene network integrating modules and pathways. R. Soc. Open Sci. 6 (7), 190214. 10.1098/rsos.190214 31417727 PMC6689581

[B39] Mohammad F. Al Ahmadi S. Al Muhtadi J. (2023). Blockchain-based deep CNN for brain tumor prediction using MRI scans. Diagnostics 13 (7), 1229. 10.3390/diagnostics13071229 37046446 PMC10093074

[B40] Ning L. Cui T. Zheng B. Wang N. Luo J. Yang B. (2021). MNDR v3. 0: Mammal ncRNA–disease repository with increased coverage and annotation. Nucleic Acids Res. 49 (D1), D160–D164. 10.1093/nar/gkaa707 32833025 PMC7779040

[B41] Noman A. A. Rahaman M. Pranto T. H. Rahman R. M. (2023). Blockchain for medical collaboration: A federated learning-based approach for multi-class respiratory disease classification. Healthc. Anal. 3, 100135. 10.1016/j.health.2023.100135

[B42] Pan Y. Lei X. Zhang Y. (2022). Association predictions of genomics, proteinomics, transcriptomics, microbiome, metabolomics, pathomics, radiomics, drug, symptoms, environment factor, and disease networks: A comprehensive approach. Med. Res. Rev. 42 (1), 441–461. 10.1002/med.21847 34346083

[B43] Park D. J. Park M. W. Lee H. Kim Y. J. Kim Y. Park Y. H. (2021). Development of machine learning model for diagnostic disease prediction based on laboratory tests. Sci. Rep. 11 (1), 7567. 10.1038/s41598-021-87171-5 33828178 PMC8026627

[B44] Pathak Y. Shukla P. K. Tiwari A. Stalin S. Singh S. Shukla P. K. (2022). Deep transfer learning based classification model for COVID-19 disease. IRBM 43 (2), 87–92. 10.1016/j.irbm.2020.05.003 32837678 PMC7238986

[B45] Rahal H. R. Slatna S. Kazar O. Barka E. Harous S. (2023). Blockchain-based multi-diagnosis deep learning application for various diseases classification.

[B46] Rao S. S. (2023). Eadn: Enhanced auto encoder decoder network ensembled with boosting technique for feature selection of Parkinson’s disease detection.

[B47] Ren K. Zou K. Liu X. Chen Y. Yuan X. Shen X. (2023). Uncertainty-informed mutual learning for joint medical image classification and segmentation.

[B48] Sammut S.-J. Crispin-Ortuzar M. Chin S. F. Provenzano E. Bardwell H. A. Ma W. (2022). Multi-omic machine learning predictor of breast cancer therapy response. Nature 601 (7894), 623–629. 10.1038/s41586-021-04278-5 34875674 PMC8791834

[B49] Sandya V. Baligeri V. Lal B. Petli V. Pradeep Kumar S. (2023). “Deep learning based brain tumor detection with internet of things,” in Proceeding of the 2023 IEEE International Conference on Integrated Circuits and Communication Systems (ICICACS), Raichur, India, February 2023 (IEEE), 1–6.

[B50] Seol S. Kwon J. Kang H. J. (2023). Cell type characterization of spatiotemporal gene co-expression modules in Down syndrome brain. iScience 26 (1), 105884. 10.1016/j.isci.2022.105884 36647384 PMC9840153

[B51] Sharma S. Guleria K. (2023). A deep learning based model for the detection of Pneumonia from chest X-ray images using VGG-16 and neural networks. Procedia Comput. Sci. 218, 357–366. 10.1016/j.procs.2023.01.018

[B52] Shi H. Zhang X. Tang L. Liu L. (2022). Heterogeneous graph neural network for lncRNA-disease association prediction. 10.1038/s41598-022-22447-y PMC9585029 36266433

[B53] Shoaib M. Shah B. Ei-Sappagh S. Ali A. Ullah A. Alenezi F. (2023). An advanced deep learning models-based plant disease detection: A review of recent research. Front. Plant Sci. 14, 1158933. 10.3389/fpls.2023.1158933 37025141 PMC10070872

[B54] Silverman E. K. Schmidt H. H. H. W. Anastasiadou E. Altucci L. Angelini M. Badimon L. (2020). Molecular networks in network medicine: Development and applications. Wiley Interdiscip. Rev. Syst. Biol. Med. 12 (6), e1489. 10.1002/wsbm.1489 32307915 PMC7955589

[B55] Subramanian I. Verma S. Kumar S. Jere A. Anamika K. (2020). Multi-omics data integration, interpretation, and its application. Bioinforma. Biol. Insights 14, 1177932219899051. 10.1177/1177932219899051 PMC7003173 32076369

[B56] Tomczak K. Czerwińska P. Wiznerowicz M. (2015). The cancer genome atlas (TCGA): An immeasurable source of knowledge. Contemp. Oncol. Pozn. 19 (1), A68–A77. 10.5114/wo.2014.47136 25691825 PMC4322527

[B57] Vaka A. R. Soni B. K S. R. (2020). Breast cancer detection by leveraging Machine Learning. ICT Express 6 (4), 320–324. 10.1016/j.icte.2020.04.009

[B58] Volovat S. R. Volovat C. Hordila I. Hordila D. A. Mirestean C. C. Miron O. T. (2020). MiRNA and LncRNA as potential biomarkers in triple-negative breast cancer: A review. Front. Oncol. 10, 526850. 10.3389/fonc.2020.526850 33330019 PMC7716774

[B59] Wandy J. Daly R. (2021). GraphOmics: An interactive platform to explore and integrate multi-omics data. BMC Bioinforma. 22 (1), 603. 10.1186/s12859-021-04500-1 PMC8684259 34922446

[B60] Wang W. Chen H. (2022). Predicting miRNA-disease associations based on lncRNA–miRNA interactions and graph convolution networks. Briefings Bioinforma. 24, bbac495. 10.1093/bib/bbac495 36526276

[B61] Wang Z. Wang L. (2022). An attention approach for dimensionality reduction that can correct feature collection skewness. IEEE Access 10, 117273–117280. 10.1109/access.2022.3220245

[B62] Wang T. Shao W. Huang Z. Tang H. Zhang J. Ding Z. (2021). MOGONET integrates multi-omics data using graph convolutional networks allowing patient classification and biomarker identification. Nat. Commun. 12 (1), 3445. 10.1038/s41467-021-23774-w 34103512 PMC8187432

[B63] Wang M. Wei Z. Jia M. Chen L. Ji H. (2022). Deep learning model for multi-classification of infectious diseases from unstructured electronic medical records. BMC Med. Inf. Decis. Mak. 22 (1), 41. 10.1186/s12911-022-01776-y PMC8848865 35168624

[B64] Wang S. Lin B. Zhang Y. Qiao S. Wang F. Wu W. (2022). Sgaemda: Predicting miRNA-disease associations based on stacked graph autoencoder. Cells 11 (24), 3984. 10.3390/cells11243984 36552748 PMC9776508

[B65] Watanabe A. Ketabi S. Namdar K. Khalvati F. (2022). Improving disease classification performance and explainability of deep learning models in radiology with heatmap generators. Front. Radiology 2, 35. 10.3389/fradi.2022.991683 PMC10365129 37492678

[B66] Wekesa J. S. Luan Y. Chen M. Meng J. (2019). A hybrid prediction method for plant lncRNA-protein interaction. Cells 8 (6), 521. 10.3390/cells8060521 31151273 PMC6627874

[B67] Wekesa J. S. Meng J. Luan Y. (2020a). A deep learning model for plant lncRNA-protein interaction prediction with graph attention. Mol. Genet. Genomics 295 (5), 1091–1102. 10.1007/s00438-020-01682-w 32409904

[B68] Wekesa J. S. Meng J. Luan Y. (2020b). Multi-feature fusion for deep learning to predict plant lncRNA-protein interaction. Genomics 112 (5), 2928–2936. 10.1016/j.ygeno.2020.05.005 32437848

[B69] Winkle M. El-Daly S. M. Fabbri M. Calin G. A. (2021). Noncoding RNA therapeutics — Challenges and potential solutions. Nat. Rev. Drug Discov. 20 (8), 629–651. 10.1038/s41573-021-00219-z 34145432 PMC8212082

[B70] Wu Z. Basu S. Wu X. Kurgan L. (2023). qNABpredict: quick, accurate and taxonomy-aware sequence-based prediction of content of nucleic acid binding amino acids. Protein Sci. 32, e4544. 10.1002/pro.4544 36519304 PMC9798252

[B71] Xie C. Zhu H. Fei Y. Yang X. Wang X. Meng Y. (2022). Interleukin-17A mediates tobacco smoke-induced lung cancer epithelial-mesenchymal transition through transcriptional regulation of ΔNp63α on miR-19. IET Image Process. 16 (1), 273–289. 10.1007/s10565-021-09594-0 33811578

[B72] Xie X. Wang Y. Sheng N. Zhang S. Cao Y. Fu Y. (2022). Predicting miRNA-disease associations based on multi-view information fusion. Front. Genet. 13, 979815. 10.3389/fgene.2022.979815 36238163 PMC9552014

[B73] Xuan P. Cao Y. Zhang T. Kong R. Zhang Z. (2019). Dual convolutional neural networks with attention mechanisms based method for predicting disease-related lncRNA genes. Front. Genet. 10, 416. 10.3389/fgene.2019.00416 31130990 PMC6509943

[B74] Yang M. Yang Y. Xie C. Ni M. Liu J. Yang H. (2022). Contrastive learning enables rapid mapping to multimodal single-cell atlas of multimillion scale. Nat. Mach. Intell. 4 (8), 696–709. 10.1038/s42256-022-00518-z

[B75] Yu Z. Amin S. U. Alhussein M. Lv Z. (2021). Research on disease prediction based on improved DeepFM and IoMT. IEEE Access 9, 39043–39054. 10.1109/access.2021.3062687

[B76] Yu H. Shen Z. A. Zhou Y. K. Du P. F. (2022). Recent advances in predicting protein-lncRNA interactions using machine learning methods. Curr. Gene Ther. 22 (3), 228–244. 10.2174/1566523221666210712190718 34254917

[B77] Yu Z. Wang K. Wan Z. Xie S. Lv Z. (2022). Popular deep learning algorithms for disease prediction: A review. Clust. Comput. 26, 1231–1251. 10.1007/s10586-022-03707-y PMC9469816 36120180

[B78] Zhang S. He X. Zhang R. Deng W. (2021). LncR2metasta: A manually curated database for experimentally supported lncRNAs during various cancer metastatic events. Brief. Bioinform 22 (3), bbaa178. 10.1093/bib/bbaa178 32766766

[B79] Zhang S. Jiang H. Gao B. Yang W. Wang G. (2022). Identification of diagnostic markers for breast cancer based on differential gene expression and pathway network. Front. Cell. Dev. Biol. 9, 811585. 10.3389/fcell.2021.811585 35096840 PMC8790293

[B80] Zhao Z. Wang K. Wu F. Wang W. Zhang K. Hu H. (2018). circRNA disease: a manually curated database of experimentally supported circRNA-disease associations. Cell. Death Dis. 9 (5), 475. 10.1038/s41419-018-0503-3 29700306 PMC5919922

[B81] Zhao Y. Wang S. Ren Y. Zhang Y. (2022). CRANet: A comprehensive residual attention network for intracranial aneurysm image classification. BMC Bioinforma. 23 (1), 322. 10.1186/s12859-022-04872-y PMC9356401 35931949

[B82] Zhu X. Shi J. Lu C. (2019). Cloud health resource sharing based on consensus-oriented blockchain technology: Case study on a breast tumor diagnosis service. J. Med. Internet Res. 21 (7), e13767. 10.2196/13767 31339106 PMC6683652

